# Heart failure hospitalization in patients with and without type 2 diabetes: A population-based retrospective cohort study

**DOI:** 10.1371/journal.pone.0351763

**Published:** 2026-07-02

**Authors:** Allison K. Shay, Wei Tao, Soumya Patnaik, Delaney K. Sullivan, Angelo Nascimbene, Xueying Wang, Joseph B. McCormick, Hulin Wu

**Affiliations:** 1 St. George’s University School of Medicine, St. George’s, Grenada; 2 Department of Biostatistics and Data Science, School of Public Health, University of Texas Health Science Center at Houston, Houston, Texas, United States of America; 3 Department of Internal Medicine, Cardiology Division, McGovern Medical School, University of Texas Health Science Center at Houston, Houston, Texas, United States of America; 4 UCLA-Caltech Medical Scientist Training Program, David Geffen School of Medicine, University of California, Los Angeles, California, United States of America; 5 Department of Epidemiology, University of Texas School of Public Health, Brownsville, Texas, United States of America; Faculdade de Medicina de São José do Rio Preto, BRAZIL

## Abstract

**Purpose:**

Type 2 diabetes mellitus (T2DM) is a common comorbidity among patients with heart failure (HF). This study aims to describe how T2DM relates to HF hospitalization patterns across demographic groups and HF subtypes.

**Methods:**

We conducted a population-based retrospective cohort study using 19 years of clinical data from Cerner Health Facts^®^, a nationwide electronic health record (EHR) database. Adult patients hospitalized with HF were identified using ICD-9/ICD-10 diagnosis codes and HF-related medications and were stratified by T2DM status. Measures included HF-related hospitalization count, length of first HF hospitalization, and age at first HF hospitalization. Comparisons were examined across HF subtypes (historically termed systolic, diastolic, other). Additionally, sensitivity analyses were conducted using alternative inclusion criteria to assess the robustness of the findings.

**Results:**

We identified 137,785 HF patients from the EHR database, among whom 29.5% had T2DM. Overall HF hospitalization was more common in men than in women; however, diastolic HF was more prevalent among women and presented at older ages whereas systolic HF was more prevalent among men. Compared with patients without T2DM, patients with T2DM experienced higher HF hospitalization count (mean: 2.81 vs. 2.42, p < 0.001), and both longer stay (mean: 7.03 vs. 6.85 days, p < 0.001) and earlier age (mean: 68.9 vs. 70.4 years, p < 0.001) of initial HF hospitalization. Across HF subtypes, T2DM was more prevalent among patients with diastolic HF (31.4% diastolic vs. 29.5% systolic vs. 28.7% other), and patients with T2DM were younger at first HF hospitalization than those without T2DM, with the largest difference observed for diastolic HF (mean: 70.4 vs. 73.1 years, p < 0.001).

**Conclusions:**

In this large nationwide EHR cohort, T2DM was associated with more intensive HF hospitalization patterns and was more prevalent in the diastolic HF subtype. These findings highlight the relevance of diabetes status and HF subtype, in addition to demographic factors, in shaping HF-related healthcare utilization.

## Introduction

Heart failure (HF) is one of the leading causes for hospitalization in the United States. The Centers for Disease Control and Prevention (CDC) estimates that cardiovascular disease, including HF, is largely preventable but costs approximately $1 billion per day [1]. Type 2 diabetes mellitus (T2DM) is a major risk factor for the development and progression of HF and is highly prevalent in both systolic HF and diastolic HF patients [2,3]. Multiple pathophysiologic mechanisms have been proposed to explain the association between T2DM and HF, including chronic hyperglycemia-induced myocardial fibrosis, microvascular dysfunction, systemic inflammation, altered myocardial energetics, and insulin resistance-related lipotoxicity [4]. In addition, T2DM is frequently accompanied by hypertension, obesity, and renal dysfunction, further amplifying HF risk and influencing disease trajectory [5,6]. Prior research has shown that HF patients with T2DM experience worse clinical outcomes [7–9]. However, less explored are the hospitalization patterns that differ among HF patients based on T2DM status. To investigate this relationship, we leveraged a real-world nationwide electronic health records (EHR) database, Cerner Health Facts^®^. This database is a comprehensive electronic health records (EHR) database that includes de-identified information on over 69 million unique patients and 380 million medical service encounters. The primary HF cohort was identified using ICD-9/ICD-10 diagnosis codes for HF as well as the requirement for HF medication. Additional cohorts were constructed using alternative inclusion criteria and HF definitions. These additional cohorts were used in sensitivity analyses to confirm the results obtained from the primary HF cohort. Of additional note, while updated guidelines classify HF based on ejection fraction (EF) as HFrEF (HF with reduced EF), HFmEF (HF with moderately reduced EF), and HFpEF (HF with preserved EF), this study uses the older classification system—where systolic HF refers to HFrEF and diastolic HF refers to HFpEF, without an intermediate HFmEF category—because the ICD-9/ICD-10 diagnosis codes used in the EHR database are based on this earlier framework [10].

## Materials and methods

### Study design

We acquired the Cerner Health Facts^®^, a multi-ethnic, de-identified, HIPAA-compliant, IRB-approved, nationwide electronic medical record database for research purposes. The Cerner EHR database contains healthcare records from 85 healthcare systems with 750 facilities across the United States from 2000 to 2018, which includes longitudinal visits with detailed records of diagnoses, medications, procedures, and lab tests, representing a total of 69 million patients across the United States. Of the 69 million patients, 55.07% were women: with 53.38% White, 11.94% African American, and 34.68% other races. In total, the database includes 939 million diagnoses coded in ICD-9/ICD-10 codes, 674 million medication records, 5.3 billion clinical events in both the outpatient and inpatient setting, and 4.2 billion lab tests.

### Data extraction and heart failure cohort definition

**Fig 1:**
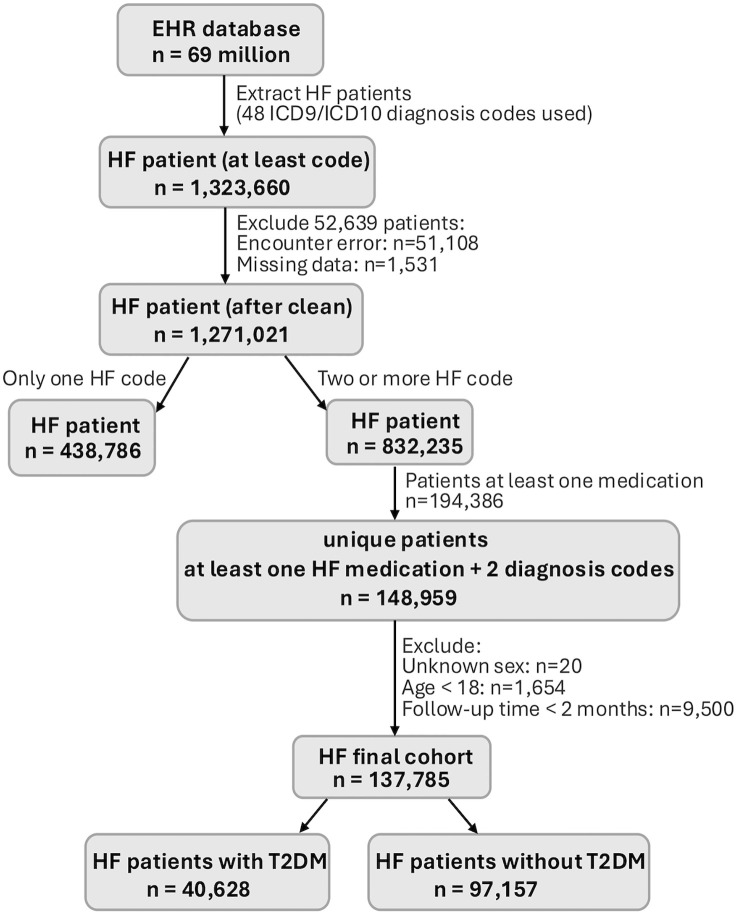
Flow chart of cohort selection.

We identified potential participants for this study among patients diagnosed with HF between 2000 and 2018 in the Cerner EHR database. Our criteria for HF patients involved having two or more ICD-9/ICD-10 HF diagnosis codes and at least one HF medication record. Because the study period spanned the national transition from ICD-9 to ICD-10, both coding systems are present in the dataset. As the cohort definitions were originally constructed using ICD-9 codes, a complete list of ICD-9 HF codes is provided in S1 Table. We retrieved data from patients with at least one of the diagnosis codes, resulting in a sample of 1,323,660 patients. After excluding those with encounter errors (n = 51,108) and missing data (n = 1,531), we were left with 1,271,021 patients. Among them, 832,235 patients had two or more HF diagnosis codes. Within this group, 194,386 patients had at least one HF medication. Furthermore, 148,959 patients met the criteria of having both at least one HF medication and two or more HF diagnosis codes. The index date for HF was defined as the date of the first HF diagnosis. We excluded patients who (a) were under 18 years old at the index visit (n = 1,654), (b) had unknown sex (n = 20), and (c) had a total follow-up time of less than 60 days (n = 9,500). The three exclusion criteria were not mutually exclusive. In total, our final data analysis included 137,785 patients (Fig 1). Among these patients, we identified those with T2DM using the eMERGE algorithm as done in our previous work [11]. eMERGE requires an HbA1c laboratory test result >6.5%, at least one ICD-9/ICD-10 T2DM diagnosis code, and prescription of at least one antihyperglycemic medication.

### Study outcomes and patient characteristic factors

The primary endpoints included length of hospital stay (LOS) during the first HF hospitalization (defined as an inpatient HF encounter lasting >24 hours and <100 days), age at first HF diagnosis (or first HF hospitalization), and the number of HF hospitalizations. Secondary endpoints included the total number of hospitalizations (including both HF-related and non-HF hospitalizations) and the frequency of all inpatient and outpatient encounters. Analyses additionally incorporated patient characteristics obtained from the Cerner electronic health record database, including age at index encounter, sex, race, and total follow-up time from the first recorded encounter to the last recorded encounter.

### Statistical methods

For EHR data processing and cleaning, we followed the guidelines developed previously by our team. The baseline characteristics were reported by percentages for categorical variables and means ± standard deviation (SD) for continuous variables. The Welch’s t-test was applied to compare differences in quantitative metrics between with/without T2DM groups while the chi-square test was applied to compare T2DM prevalence between groups. P-value < 0.05 for a two-tailed test was considered statistically significant and multiple testing adjustment was performed via the Bonferroni correction. While hospitalization counts are likely right-skewed, the large sample size justifies the use of t-tests for comparing mean differences, as the sampling distribution of the mean is approximately normal by the central limit theorem. All statistical analyses were carried out using statistical software SAS 9.4 and R.

### Manuscript writing

The OpenAI tool, ChatGPT, was used to refine grammar.

### Ethics

This study complies with the Declaration of Helsinki. The use of de-identified data from the Cerner Health Facts® database received written approval by the Institutional Review Board (IRB) of the University of Texas Health Science Center. Given the nature of this study and exclusive use of existing records that were fully de-identified prior to investigator access, the IRB waived informed consent. Data access date: June 17, 2019.

## Results

### Cohort characteristics

The final study cohort consisted of 137,785 HF patients from the Cerner Health Facts EHR database. Among the final cohort, 40,628 (29.49%) were with T2DM. Table 1 lists the summarized statistics of baseline demographic factors and patient characteristics for the HF cohort with/without T2DM, with comparisons to the overall population in the Cerner EHR database. HF prevalence increased sharply with age as expected; more than 73% were among the patients at least 60 years. Of all HF patients, the HF rate was higher (>51%) in men than women, while the proportion of men was much lower (45%) in the overall database. The HF rate was higher among African American individuals (20%) and White individuals (75%) compared to their proportions in the general Cerner database, 12% and 53%, respectively. In contrast, the HF rate in other races was much lower (5%) than their proportion (35%) in the Cerner database. HF rate was highest in White individuals overall. The average encounters per patient for HF patients was much larger (36) than that (7) for the general population in the database. The total follow-up time for HF patients was also 5.3 years, compared to 1.9 years for the general population in the database. The HF rate in patients with T2DM was lower (71%) than those without T2DM (76%) among White patients only. Among HF patients, the average number of encounters per patient and total follow-up time were larger for those patients with T2DM compared to those without T2DM (40.6 vs. 34.6 encounters and 5.5 vs. 4.2 years, respectively). Altogether, the HF patients differ markedly from the general EHR population in age, demographic composition, and healthcare utilization, with further variation between those with and without T2DM.

**Table 1:** Baseline demographic factors and patient characteristics for the HF cohort with/without T2DM.

Features		Cerner EHR database (n = 69,025,360)	All HF patients(n = 137,785)	HF with T2DM(n = 40,628)	HF without T2DM(n = 97,157)
**Age(number, %)**	18–40	19,721,291 (38.34%)	5,349(3.88%)	986(2.43%)	4,363(4.49%)
	41–60	17,468,377(33.96%)	31,006(22.50%)	9,789(24.09%)	21,217(21.84%)
	60+	14,248,754(27.70%)	101,430(73.61%)	29,853(73.48%)	71,577(73.67%)
**Sex(number, %)**	Male	29,202,345(44.93%)	70,565(51.21%)	21,007(51.71%)	49,558(51.01%)
	Female	35,793,681(55.07%)	67,220(48.79%)	19,621(48.29%)	47,599(48.99%)
**Race(number, %)**	African American	8,239,573(11.94%)	27,318(19.83%)	9,160(22.55%)	18,158(18.69%)
	White	36,833,443(53.38%)	102,947(74.72%)	28,709(70.66%)	74,238(76.41%)
	Other races	23,931,973(34.68%)	7,520(5.46%)	2,759(6.79%)	4,761(4.90%)
**All encounter counts per patient (mean ± SD)**		6.9	36.4 ± 49.5	40.6 ± 51.6	34.6 ± 48.5
**Total follow up years per patient (mean ± SD)**		1.9	5.3 ± 2.9	5.5 ± 2.9	5.2 ± 3.0

### Hospitalization outcomes for HF inpatients with and without T2DM

HF patient hospitalization was defined as HF inpatient visits lasting between 24 hours and 100 days. Table 2 presents outcomes for HF inpatients with and without T2DM. Compared with patients without T2DM, patients with T2DM experienced more frequent HF hospitalizations (mean hospitalization count: 2.81 vs. 2.42), longer initial HF hospital stays (mean: 7.03 vs. 6.85 days), and younger age at first HF hospitalization (mean: 68.9 vs. 70.4 years). All comparisons were statistically significant.

**Table 2:** Hospitalization outcomes for HF patients with/without T2DM.

Outcomes	HF inpatient(n = 115,400)	HF inpatient With T2DM(n = 33,567)	HF inpatient Without T2DM(n = 81,833)	Mean difference [95% confidence interval]	P-value	Adjusted P-value
**Total hospitalization counts(mean ± SD)**	4.67 ± 4.91	5.10 ± 5.25	4.49 ± 4.75	0.61 [0.55, 0.67]	<.001	<.001
**HF Hospitalization counts(mean ± SD)**	2.54 ± 2.90	2.81 ± 3.27	2.42 ± 2.73	0.39 [0.35, 0.43]	<.001	<.001
**Length of first HF hospitalization (days)** **(mean ± SD)**	6.90 ± 7.22	7.03 ± 7.31	6.85 ± 7.18	0.18 [0.09, 0.27]	<.001	<.001
**Age at first HF hospitalization (years)(mean ± SD)**	69.95 ± 13.98	68.85 ± 12.55	70.40 ± 14.50	−1.55 [−1.72, −1.38]	<.001	<.001
**All encounter frequency (per year) (mean ± SD)**	7.48 ± 8.77	7.85 ± 9.15	7.33 ± 8.60	0.52 [0.41, 0.63]	<.001	<.001

### HF subtype analysis of HF patients with and without T2DM

**Fig 2:**
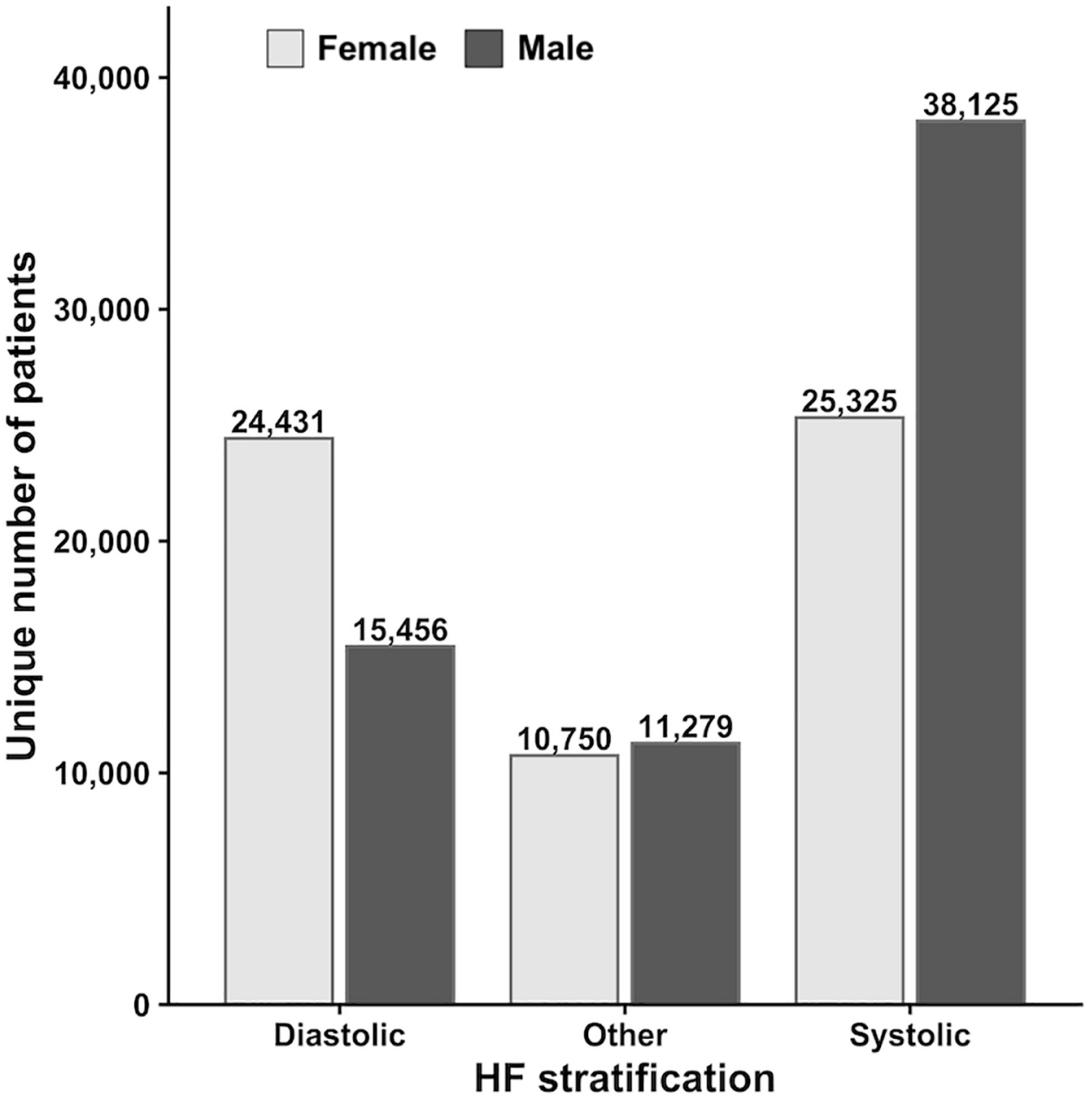
Comparison of HF subtypes by sex.

**Fig 3:**
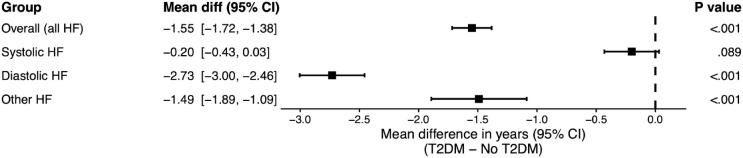
Unadjusted mean differences in age at first heart failure hospitalization by HF subtype. Points represent mean differences comparing patients with and without T2DM (T2DM minus no T2DM), and horizontal bars represent 95% confidence intervals. Negative values indicate earlier HF diagnosis among patients with T2DM.

We further divided HF into three categories, systolic, diastolic, and other subtypes based on the ICD-9/ICD-10 diagnosis codes. Patients with exclusively diastolic or exclusively systolic HF codes were classified accordingly, whereas patients without subtype-specific systolic or diastolic codes were classified as having other HF. Patients who carried both systolic and diastolic HF codes were excluded from subtype-specific analyses to avoid misclassification (12,419 patients; 9% of the HF cohort). Although overall HF hospitalization was more common among men than women (Table 1), notable sex differences emerged by HF subtype. Diastolic HF was substantially more prevalent among women, with women comprising approximately 1.6 times as many patients as men, whereas systolic HF predominated among men, who accounted for approximately 1.5 times as many patients as women (Fig 2). Across all HF subtypes, patients with T2DM were younger at first HF hospitalization compared with those without T2DM (Table 3) (Fig 3), with the largest age difference observed in diastolic HF (mean age: 70.4 vs. 73.1 years, respectively). Differences were statistically significant overall and for diastolic and “other” HF subtypes, whereas no significant difference was observed for systolic HF. In addition, patients with diastolic HF were diagnosed at older ages than those with systolic or other HF subtypes, with mean age differences ranging from 3.5 to 4.9 years. These subtype-specific patterns were preserved when analyses were restricted to HF inpatient encounters only (S3 Table); although the age difference for the systolic subtype reached statistical significance in the inpatient-only analysis, the magnitude of this difference was substantially smaller than that observed for the diastolic and “other” HF subtypes, and the overall subtype-specific trends remained consistent.

**Table 3:** Age at first diagnosis for different subtypes of HF.

Population	HF Subtypes	Overall Mean	HF with T2DM	HF without T2DM	P-value	Adjusted p-value
HF patient(n = 137,785)(mean ± SD)	Systolic (n = 63,450)	67.35 ± 14.43	67.21 ± 12.84	67.41 ± 15.05	.089	.267
	Diastolic (n = 39,887)	72.25 ± 13.54	70.38 ± 12.53	73.11 ± 13.89	<.001	<.001
	Other (n = 22,029)	68.75 ± 14.70	67.69 ± 13.17	69.18 ± 15.25	<.001	<.001

**Fig 4:**
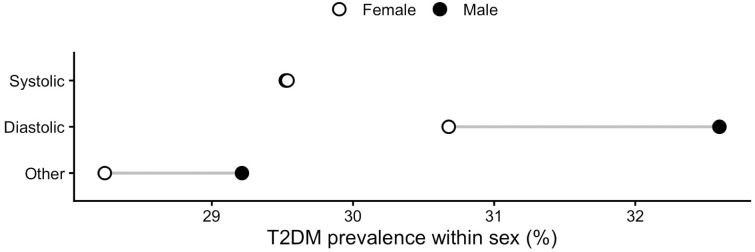
Sex-specific prevalence of type 2 diabetes mellitus across heart failure subtypes.

Table 4 summarizes the number and proportion of patients with T2DM across HF subtypes by sex. Overall, T2DM prevalence was highest among patients with diastolic HF compared with systolic and other HF subtypes (31.4% vs. 29.5% vs. 28.7%, respectively), with this pattern most pronounced among men (32.6% diastolic vs. 29.5% systolic vs. 29.2% other). Although T2DM prevalence among patients with diastolic HF and other HF subtypes was higher in men than in women, the prevalence of T2DM among patients with systolic HF was similar between sexes (Fig 4). These findings remained consistent when analyses were restricted to HF inpatient encounters only (S4 Table).

**Table 4:** T2DM and sex distributions for different subtypes of HF.

Population	HF Subtypes	Male				Female				P-value^b^	Adjusted p-value^b^
		HF with T2DM	HF without T2DM	P-value^a^	Adjusted p-value^a^	HF with T2DM	HF without T2DM	P-value^a^	Adjusted p-value^a^		
**HF patient(n = 137,785)(number,T2DM %)**	**Systolic**	11,256 (29.52%)	26,869 (70.48%)	<.001	<.001	7,480 (29.54%)	17,845 (70.46%)	<.001	<.001	.974	1
	**Diastolic**	5,038 (32.60%)	10,418 (67.40%)			7,495 (30.68%)	16,936 (69.32%)			<.001	<.001
	**Other**	3,295 (29.21%)	7,984 (70.79%)			3,036 (28.24%)	7,714 (71.76%)			.111	.555

^a^Chi-square test comparing T2DM prevalence across HF subtypes within each sex.

^b^Chi-square test comparing T2DM prevalence between sexes within each HF subtype.

### Sensitivity analyses for different definitions of HF cohort

The preceding primary analyses are based on the rigorous definition of HF cohort, i.e., the patients were included in the HF cohort if they had at least two HF-related ICD-9/ICD-10 codes and one HF-related medications (S2 Table). Sensitivity analyses were conducted using alternative, less restrictive HF definitions, and results were compared across these cohorts. Although broader HF definitions increased sample size, they may have introduced a higher likelihood of HF misclassification. Future studies would benefit from more refined EHR-based phenotyping approaches to improve HF case identification.

S5-S7 Tables summarize key sensitivity analysis results. S5 Table demonstrates consistent sex distributions across HF subtypes under different HF definitions. S6 Table shows that, across definitions, patients with T2DM were consistently younger at first HF diagnosis than those without T2DM, with the largest age differences observed among patients with diastolic HF; additionally, patients with systolic HF were younger at first diagnosis than those with diastolic HF, consistent with the primary analysis (Table 3). S7 Table indicates that although less restrictive HF definitions were associated with lower overall proportions of T2DM across subgroups, the principal patterns—higher T2DM prevalence among patients with diastolic HF compared with other HF subtypes and higher prevalence among men than women—remained consistent across HF definitions.

## Discussion

In this large, nationwide EHR-based cohort, we found that T2DM was associated with more intensive HF-related healthcare utilization and earlier HF presentation, with some variation across HF subtypes. Approximately one-third of hospitalized HF patients had T2DM, underscoring the high burden of diabetes in this population. Patients with T2DM experienced more frequent HF hospitalizations, longer initial hospital stays, and younger age at first HF hospitalization compared with patients without T2DM. Although these differences were modest in absolute magnitude, they were consistent across analyses and robust to alternative HF definitions, suggesting meaningful implications at the population level. Moreover, these findings are compatible with previous research showing poorer clinical outcomes in HF patients who have T2DM [7–9].

Consistent with prior epidemiologic studies, HF hospitalization was more common in men than in women [12–15]. However, diastolic HF was disproportionately represented among women and presented at older ages, whereas systolic HF predominated among men and presented earlier. These findings reinforce well-described sex-specific differences in HF phenotypes [16–18] and highlight the importance of considering HF subtype when evaluating demographic patterns of HF hospitalization.

A key finding of this study was the higher prevalence of T2DM among patients with diastolic HF compared with systolic and other HF subtypes, particularly among men. In addition, patients with T2DM were consistently younger at first HF hospitalization across all HF subtypes, with the largest age difference observed among those with diastolic HF. Although diastolic heart failure typically presents at older ages than other HF subtypes since it involves progressive age-related myocardial changes [19,20], T2DM may accelerate these pathophysiologic processes, leading to earlier ventricular stiffening thereby shifting diastolic heart failure presentation to an earlier age.

As mentioned previously, although the associations between T2DM and hospitalization patterns are statistically significant, their effect sizes are small but still meaningful at the system level. For example, while HF patients with T2DM have a mean of 5.10 hospitalization counts compared to 4.49 without, a difference of 0.6 additional hospitalizations still corresponds to a meaningful increase (a 14% relative increase) in healthcare utilization. While such differences may not be directly perceptible at the individual level given substantial variability, even small increases in hospitalization frequency can translate to meaningful differences in cumulative burden across populations. For hospitalization age, HF patients with T2DM have a mean age of first hospitalization of 68.85 vs. 70.40 without—a modest difference of 1.55 years which may still reflect a subtle shift toward earlier disease manifestation at the population level.

Examining racial differences in HF hospitalization patterns would be useful in future work as prior reports suggest that African American patients present with HF at younger ages than white patients [13,21]. One limitation of the present study is that only aggregate (i.e., ensemble-level) variables were examined; but the relationship between HF hospitalization pattern outcomes and T2DM status could differ depending on demographic group. The intersection of diabetes status, HF subtype, and race suggests a complex interplay of biological, socioeconomic, and healthcare access factors that influence HF presentation and utilization. Investigating these factors would help identify populations that may benefit from earlier risk stratification and targeted preventive interventions.

Electronic health records (EHR) data, despite its great utility for scientific research, faces several significant limitations. First, data quality and completeness can be inconsistent, as EHRs are primarily designed for clinical, not research, purposes. This leads to issues such as missing data, errors, and variability in how data is recorded across different clinics and hospitals. Second, the potential biases in the data sampling that arise from the non-random nature of healthcare delivery, limit the generalizability of research findings derived from EHR data. Additionally, many other confounding factors related to diabetes and HF, such as diet, physical activity, family history and genetics data are not available from the EHR database. Finally, as an observational study without confounding factor consideration, the observed associations may vary across different patient subpopulations and, additionally, causal relationships between T2DM and HF hospitalization patterns cannot be inferred. Patients with T2DM often are also burdened with other comorbidities (hypertension, chronic kidney disease, hyperlipidemia, etc.) and T2DM might not act independently in determining HF hospitalization but rather might act as a marker of higher comorbidity burden, which remains clinically informative. This study does not aim to disentangle the effects of T2DM from those of other comorbidities. Altogether, the results from this study should be interpreted and generalized with these caveats in mind.

## Conclusion

In summary, our findings are consistent with prior literature on sex-specific and subtype-specific patterns of HF and further demonstrate that T2DM is associated with earlier and more intensive HF-related healthcare utilization. These results underscore the importance of incorporating diabetes prevention and management into strategies aimed at reducing HF burden.

## Supporting information

S1 Table The number and percentage of HF patients for each of HF-related ICD-9 codes. (PDF)

S2 Table List of HF-related medications used for HF data extraction. (PDF)

S3 Table Age at first diagnosis for different subtypes of HF for HF inpatients. (PDF)

S4 Table T2DM and sex distributions for different subtypes of HF for HF inpatients. (PDF)

S5 Table The number and percentage of female patients for different HF subtypes based on different definitions of HF cohort. (PDF)

S6 Table The age (Mean ± SD by years) of first diagnosis of HF for different HF subtypes and T2DM status based on different definitions of HF cohort. (PDF)

S7 Table The number and percentage of T2DM patients for different HF subtypes among different sex based on different definitions of HF cohort. (PDF)
