# Supplementary material for: Heart failure hospitalization in patients with and without type 2 diabetes: A population-based retrospective cohort study
Source: PLoS One. 2026 Jul 2;21(7):e0351763. doi: 10.1371/journal.pone.0351763 (PMC13327123; doi:10.1371/journal.pone.0351763)
Supplement: S1 Table — (PDF) [file pone.0351763.s001.pdf]

ICD9_code	Description	Patient count n=137,785	Percent (%)
428	Heart failure	106904	77.6
428.23	Acute on chronic systolic heart failure	4232	3.07
428.22	Chronic systolic heart failure	4092	2.97
428.33	Acute on chronic diastolic heart failure	3109	2.26
428.32	Chronic diastolic heart failure	2893	2.1
428.21	Acute systolic heart failure	2888	2.1
428.3	Diastolic heart failure	2189	1.59
428.31	Acute diastolic heart failure	2101	1.53
428.2	Systolic heart failure, unspecified	1585	1.15
428.9	Heart failure, unspecified	1381	1
428.43	Acute on chronic combined systolic and diastolic heart failure	1204	0.87
402.91	Unspecified hypertensive heart disease with heart failure	1076	0.78
428.1	Left heart failure	1027	0.75
428.42	Chronic combined systolic and diastolic heart failure	624	0.45
404.91	Hypertensive heart and chronic kidney disease, unspecified, with heart failure and with chronic kidney disease stage I through stage IV, or unspecified	581	0.42
428.41	Acute combined systolic and diastolic heart failure	505	0.37
398.91	Rheumatic heart failure (congestive)	406	0.29
428.4	Combined systolic and diastolic heart failure	277	0.2
402.11	Benign hypertensive heart disease with heart failure	250	0.18
404.93	Hypertensive HF and CKD-Kidney Failure	165	0.12
404.01	Hypertensive heart and chronic kidney disease, malignant, with heart failure and with chronic kidney disease stage I through stage IV, or unspecified	119	0.09
404.11	Hypertensive heart and chronic kidney disease, benign, with heart failure and with	71	0.05

	chronic kidney disease stage I through stage IV, or unspecified		
404.13	Hypertensive heart and chronic kidney disease, benign, with heart failure and chronic kidney disease stage V or end stage renal disease	40	0.03
404.03	Hypertensive heart and chronic kidney disease, malignant, with heart failure and with chronic kidney disease stage V or end stage renal disease	38	0.03
402.1	Benign hypertensive heart disease	8	0.01
404.1	Benign hypertensive heart and renal disease	1	0
