# Supplementary material for: Heart failure hospitalization in patients with and without type 2 diabetes: A population-based retrospective cohort study
Source: PLoS One. 2026 Jul 2;21(7):e0351763. doi: 10.1371/journal.pone.0351763 (PMC13327123; doi:10.1371/journal.pone.0351763)
Supplement: S2 Table — (PDF) [file pone.0351763.s002.pdf]

Medications: Generic name (trade name)
Captopril (Capoten), Enalapril (Vasotec), Fosinopril (Monopril), Lisinopril (Prinivil, Zestril), Perindopril (Aceon), Quinapril (Accupril), Ramipril (Altace), Trandolapril (Mavik), Moexipril (Univasc), Candesartan (Atacand), Losartan (Cozaar), Valsartan (Diovan), Sacubitril/valsartan, Ivabradine (Corlanor), Bisoprolol (Zebeta), Metoprolol succinate (Toprol XL), Carvedilol (Coreg), Carvedilol CR (Coreg CR)Toprol XL, Spironolactone (Aldactone), Eplerenone (Inspra), Hydralazine and isosorbide dinitrate (combination drug) - (Bidil), Furosemide (Lasix), Bumetanide (Bumex), Torsemide (Demadex), Chlorothiazide (Diuril), Amiloride (Midamor Chlorthalidone (Hygroton), Hydrochlorothiazide or HCTZ (Esidrix, Hydrodiuril), Indapamide (Lozol), Metolazone (Zaroxolyn), Triamterene (Dyrenium)
