# Supplementary material for: Heart failure hospitalization in patients with and without type 2 diabetes: A population-based retrospective cohort study
Source: PLoS One. 2026 Jul 2;21(7):e0351763. doi: 10.1371/journal.pone.0351763 (PMC13327123; doi:10.1371/journal.pone.0351763)
Supplement: S3 Table — (PDF) [file pone.0351763.s003.pdf]

| Population | HF Types | Overall Mean | HF with T2DM | HF without T2DM | P-Value |
|---|---|---|---|---|---|
| HF inpatient (n=115,400) (mean ± SD) | Systolic (n=56,224) | 67.77 ± 14.20 | 67.39 ± 12.65 | 67.93 ± 14.79 | <0.001 |
| | Diastolic (n=34,602) | 73.06 ± 12.84 | 70.95 ± 11.99 | 73.99 ± 13.09 | <0.001 |
| | Other (n=16,184) | 69.51 ± 14.35 | 68.22 ± 12.88 | 70.02 ± 14.87 | <0.001 |
