# Supplementary material for: Heart failure hospitalization in patients with and without type 2 diabetes: A population-based retrospective cohort study
Source: PLoS One. 2026 Jul 2;21(7):e0351763. doi: 10.1371/journal.pone.0351763 (PMC13327123; doi:10.1371/journal.pone.0351763)
Supplement: S4 Table — (PDF) [file pone.0351763.s004.pdf]

Population	HF Types	Male		Female	
		HF with T2DM	HF without T2DM	HF with T2DM	HF without T2DM
HF inpatient (n=115,400) (number, T2DM %)	Systolic	9,934 (29.23%)	24,049 (70.77%)	6,476 (29.12%)	15,765 (70.88%)
	Diastolic	4,224 (31.75%)	9,079 (68.25%)	6,342 (29.78%)	14,957 (70.22%)
	Other	2,447 (29.08%)	5,967 (70.92%)	2,182 (28.08%)	5,588 (71.92%)
