# Supplementary material for: Heart failure hospitalization in patients with and without type 2 diabetes: A population-based retrospective cohort study
Source: PLoS One. 2026 Jul 2;21(7):e0351763. doi: 10.1371/journal.pone.0351763 (PMC13327123; doi:10.1371/journal.pone.0351763)
Supplement: S5 Table — (PDF) [file pone.0351763.s005.pdf]

Sensitivity analysis results for different definitions of HF cohort

Some of the primary analyses were repeated for four different definitions of HF cohort: 1) One or more HF-related ICD-9 codes (n=1,271,021); 2) at least two or more HF-related ICD-9 codes (n=832,235); 3) at least one HF-related ICD-9 codes and one HF-related medication (n=194,386); and 4) at least two HF-related codes and one HF-related medications (n=137,785), which is the definition we used for our primary data analyses. Selected results are shown below.

HF Types	At least one HF code n=1,271,021	Two or more HF codes n=832,235	one HF code +one medication n=194,386	two HF codes +one medication n=137,785
Systolic	104,930 (40.34%)	102,228 (40.40%)	25,620 (39.93%)	25,325 (39.91%)
Diastolic	134,770 (61.46%)	129,400 (61.51%)	27,936 (60.64%)	24,431 (61.25%)
Other	73,675 (50.99%)	65,982 (51.04%)	18,975 (48.87%)	10,750 (48.80%)
