# Supplementary material for: Heart failure hospitalization in patients with and without type 2 diabetes: A population-based retrospective cohort study
Source: PLoS One. 2026 Jul 2;21(7):e0351763. doi: 10.1371/journal.pone.0351763 (PMC13327123; doi:10.1371/journal.pone.0351763)
Supplement: S6 Table — (PDF) [file pone.0351763.s006.pdf]

Sensitivity analysis results for different definitions of HF cohort

Some of the primary analyses were repeated for four different definitions of HF cohort: 1) One or more HF-related ICD-9 codes (n=1,271,021); 2) at least two or more HF-related ICD-9 codes (n=832,235); 3) at least one HF-related ICD-9 codes and one HF-related medication (n=194,386); and 4) at least two HF-related codes and one HF-related medications (n=137,785), which is the definition we used for our primary data analyses. Selected results are shown below.

T2DM Status	HF Types	At least one HF code n=1,271,021	Two or more HF codes n=832,235	one HF code + one medication n=194,386	two HF codes + one medication n=137,785
All	Systolic HF	68.77 ± 14.58	68.80 ± 14.56	67.37 ± 14.44	67.35 ± 14.43
	Diastolic HF	72.90 ± 13.65	72.99 ± 13.61	72.25 ± 13.53	72.25 ± 13.54
	Other HF	69.33 ± 15.05	69.74 ± 14.86	68.66 ± 14.73	68.75 ± 14.70
HF with T2DM	Systolic HF	68.44 ± 12.76	68.43 ± 12.76	67.21 ± 12.84	67.21 ± 12.84
	Diastolic HF	71.05 ± 12.43	71.10 ± 12.41	70.36 ± 12.54	70.38 ± 12.53
	Other HF	68.73 ± 13.07	68.81 ± 13.08	67.66 ± 13.17	67.69 ± 13.17
HF without T2DM	Systolic HF	68.90 ± 15.15	68.93 ± 15.13	67.44 ± 15.05	67.41 ± 15.05
	Diastolic HF	73.54 ± 14.00	73.65 ± 13.95	73.11 ± 13.88	73.11 ± 13.89
	Other HF	69.48 ± 15.51	69.99 ± 15.30	69.06 ± 15.29	69.18 ± 15.25
