# Supplementary material for: Heart failure hospitalization in patients with and without type 2 diabetes: A population-based retrospective cohort study
Source: PLoS One. 2026 Jul 2;21(7):e0351763. doi: 10.1371/journal.pone.0351763 (PMC13327123; doi:10.1371/journal.pone.0351763)
Supplement: S7 Table — (PDF) [file pone.0351763.s007.pdf]

Sensitivity analysis results for different definitions of HF cohort

Some of the primary analyses were repeated for four different definitions of HF cohort: 1) One or more HF-related ICD-9 codes (n=1,271,021); 2) at least two or more HF-related ICD-9 codes (n=832,235); 3) at least one HF-related ICD-9 codes and one HF-related medication (n=194,386); and 4) at least two HF-related codes and one HF-related medications (n=137,785), which is the definition we used for our primary data analyses. Selected results are shown below.

Sex	HF Types	At least one HF code n=1,271,021	Two or more HF codes n=832,235	one HF code + One medication n=194,386	two HF codes + one medication n=137,785
Female	Systolic	26,566 (25.32%)	26,173 (25.60%)	7,528 (29.38%)	7,480 (29.54%)
	Diastolic	33,581 (24.92%)	32,668 (25.25%)	7,585 (30.51%)	7,495 (30.68%)
	Other	14,738 (20.00%)	13,696 (20.76%)	3,129 (27.85%)	3,036 (28.24%)
Male	Systolic	39,894 (25.71%)	39,187 (25.98%)	11,338 (29.41%)	11,256 (29.52%)
	Diastolic	22,549 (26.69%)	21,907 (27.05%)	5,097 (32.40%)	5,038 (32.60%)
	Other	15,017 (21.21%)	13,926 (22.00%)	3,416 (28.84%)	3,295 (29.21%)
