# Supplementary material for: Heart failure hospitalization in patients with and without type 2 diabetes: A population-based retrospective cohort study
Source: PLoS One. 2026 Jul 2;21(7):e0351763. doi: 10.1371/journal.pone.0351763 (PMC13327123; doi:10.1371/journal.pone.0351763)
Supplement: Attachment — Submitted filename: Response To Reviewers.pdf [file pone.0351763.s008.pdf]

We note that you have indicated that there are restrictions to data sharing for this study. For studies involving human research participant data or other sensitive data, we encourage authors to share de-identified or anonymized data. However, when data cannot be publicly shared for ethical reasons, we allow authors to make their data sets available upon request. For information on unacceptable data access restrictions, please see http://journals.plos.org/plosone/s/data-availability#loc-unacceptable-data-access-restrictions.

Before we proceed with your manuscript, please address the following prompts:

a) If there are ethical or legal restrictions on sharing a de-identified data set, please explain them in detail (e.g., data contain potentially identifying or sensitive patient information, data are owned by a third-party organization, etc.) and who has imposed them (e.g., a Research Ethics Committee or Institutional Review Board, etc.). Please also provide contact information for a data access committee, ethics committee, or other institutional body to which data requests may be sent.

b) If there are no restrictions, please upload the minimal anonymized data set necessary to replicate your study findings to a stable, public repository and provide us with the relevant URLs, DOIs, or accession numbers. Please see http://www.bmj.com/content/340/bmj.c181.long for guidelines on how to de-identify and prepare clinical data for publication. For a list of recommended repositories, please see https://journals.plos.org/plosone/s/recommended-repositories. You also have the option of uploading the data as Supporting Information files, but we would recommend depositing data directly to a data repository if possible.

Please update your Data Availability statement in the submission form accordingly.

There are legal and ethical restrictions on sharing the CERNER dataset. The CERNER database is already de-identified and HIPAA-compliant. However, like most other databases containing sensitive patient records (even when HIPAA-compliant), it is a private dataset and requires a data use agreement to access. The data itself is owned by Cerner Corporation (now Oracle Health). As such, uploading the dataset either in part or in full would not be feasible. All requests for access to this dataset must be obtained via request directly to Oracle Health. None of the authors had any special access privileges to the data and access was obtained through request of a data use agreement.

The data availability statement has been updated to clarify this.

Reviewer #1: Shay et al. present a population-based retrospective cohort study on the effects of comorbidities particularly T2DM on the pattern and frequency of hospitalization in patients with heart failure. The authors leveraged data from a real-world nationwide electronic health records (EHR) database to identify patients with HF diagnosis based on ICD diagnosis codes and use of HF medication. The authors found that compared with patients without T2DM, patients with T2DM experienced more frequent HF hospitalizations, longer initial HF hospital stays, and younger age at first HF hospitalization. By performing a HF subtype analysis of HF patients with and without T2DM, the authors found that diastolic HF was substantially more prevalent among women, whereas systolic HF predominated among men. Also, they found that patients with diastolic HF were diagnosed at older ages than those with systolic or other HF subtypes. In addition, the authors reported higher prevalence of T2DM in patients with diastolic HF compared with systolic and other HF subtypes. Furthermore, the authors performed sensitivity analysis using a replication cohort defined by a less restrictive HF definition.
Overall, the manuscript is clear; the data and findings are presented in a lucid manner.

Major issues:
- Given that the patient cohort used for the analysis most likely have other comorbidities which can confound the results, it would be important to adjust for this in the analysis. It would be great if this could be addressed.

We agree that the patient cohort will likely have other comorbidities (patients with T2DM often are also burdened with hypertension, chronic kidney disease, hyperlipidemia, etc.). However, the primary objective of this study was to provide a descriptive, population-level characterization of HF hospitalization patterns across T2DM status, rather than to estimate the independent causal effect of T2DM. As such, unadjusted comparisons rather than full multivariable adjustment for all comorbid conditions were used to reflect real-world differences between patient groups.

From a clinical perspective, T2DM often does not occur in isolation but rather as part of a broader cardiometabolic phenotype and the observed associations reflect the real-world risk profile of patients with T2DM. Regardless of whether T2DM acts independently or as a marker of higher comorbidity burden, it remains a useful indicator of HF hospitalization risk.

Thus, we believe that the importance and clinical utility of our findings remain intact, while leaving interesting directions for future work.

We clarify this further in the discussion of the revised manuscript.

- Adjustment for multiple testing - the statistics was not adjusted for multiple testing. This is important regardless of the exploratory nature of the analysis. Kindly provide adjusted p-values for the various comparisons.

We have now added Bonferroni adjusted p-values throughout the manuscript.

- Tables 3 & 4 lack p-value column. Kindly update to include raw and adjusted p-values.

We thank the reviewer for bringing this to our attention. We have added the raw and adjusted p-values to the tables.

Reviewer #2: The authors performed an elegant retrospective analysis of a cohort of patients with heart failure. Among the main findings, the higher rate of hospitalizations in T2DM patients stands out. The results are well presented, and the statistical methodology is appropriate. The authors adequately report the limitations of this type of analysis.

We thank the reviewer for the feedback, and we are grateful to the reviewer for taking the time to review our work.

Reviewer #3: Reviewer comments on the Manuscript: PONE-D-26-0028
This study analyzes 137,785 heart failure (HF) patients from a nationwide electronic health record (EHR) database to evaluate differences in hospitalization patterns between patients with and without type 2 diabetes mellitus (T2DM). The authors report higher hospitalization counts in T2DM patients, slightly longer length of stay, and younger age at first HF hospitalization, higher prevalence of T2DM in diastolic HF. The paper is a solid observational paper with several strengths such as large sample size, based on a nationwide real-world dataset and a clinically relevant topic. Nonetheless, there are some methodological concerns:
Major revision
1) In multivariate analyses there is a statement that multiple testing adjustment was not performed. Considering the large sample size, It will be important to adjust for age, sex, race, follow-up time in the cohort, comorbidities and healthcare access or utilization in parallel. The authors should provide adjustment for the primary outcomes considered: LOS, age ta hospitalization, and cost.

Firstly, we extend our deepest thanks to the reviewer for a careful review of our manuscript and for taking an interest in our work.

We have now performed multiple hypothesis testing adjustment. However, a full parallel multivariate adjustment of other factors is outside the scope of the present study and would require a full, dedicated re-analysis of the data. We do acknowledge these factors at play and this point is highlighted as a key limitation of the study. We believe that, despite these acknowledged limitations, the study in its current form, with the manuscript revisions made, is appropriate for consideration under the methodological and reporting standards of PLOS ONE.

2) Although the results as statically significant their clinical impact is probably small. This should be addressed in the paper. Or translate into more relevant impact, if identified.

We thank the reviewer for this feedback. It is indeed important to contrast effect size and statistical significance. We have now added effect sizes in our discussion. Our effect sizes are seemingly small but still relevant and expected. They are more useful at the system-level / population-level rather than at the individual patient level and we have clarified this point by adding an additional paragraph in the discussion portion of the text.

3) No definition of diabetes severity was reported. Important to consider duration, HbA1c levels, insulin use, complications. Most of them could have had a significant effect on the results and be important to discuss.

This reviewer astutely points out that we did not actually define how we identified T2DM patients in our cohort. We apologize for this oversight. Our identification algorithm mirrors that of our previous work [1]. We have now added this citation as well as the T2DM-identification algorithm (eMERGE) to our paper.

[1] Brady et al., 2022, Diabetes Spectr - https://pmc.ncbi.nlm.nih.gov/articles/PMC9160545/ - "To exclude people with type 1 diabetes and other diabetes-related diseases from actual type 2 diabetes cases, eMERGE requires an A1C laboratory test result >6.5%, at least one type 2 diabetes diagnosis code, and prescription of at least one antihyperglycemic medication."

4) Statistical analyses should be reviewed accounting for multiple outcomes, comparisons and subgroups. . In addition, the number of hospitalizations probably is not normal and skewed, important to adjust in regression, provide effect sizes and confidence intervals.

We have added adjustments for multiple outcome testing via Bonferroni correction.

While hospitalization counts are likely right-skewed, the large sample size allows for robust estimation of mean differences via the central limit theorem. We have added this point to the text.

As an aside, we additionally note (now in the updated text) that that the t-test performed throughout the paper is actually Welch's t-test, not the standard Student's t-test. (Welch's t-test is preferable in our study given the large difference in sample sizes between groups).

A good point brought up by the reviewer is to provide effect sizes and confidence intervals for number of hospitalizations – we have now included this in Table 2.

5) Review the concept of HF phenotypes, according to international guidelines, patients are classified as HF with preserved, mid-range, or reduced EF. Diastolic is no longer used to identified these population. In addition some etiology or mechanistic aspects could be explored in the data base to better understand the results. For example, patients with prior MI or angioplasty or microvascular disease had worse outcomes.

This is another really good point. Unfortunately, since the dataset uses ICD codes and exact EF values aren't readily available to us, we had to stick with this outdated and imprecise nomenclature and were unable to reclassify patients according to guideline-defined EF categories. That said, we now mention this nuance in the paper (and include a citation to the 2021 ESC guidelines in the introduction) as we believe it is important.

Exploring mechanistic aspects is intriguing and would introduce significant novelty to this work; however, as in our response to reviewer #1, is outside of the scope of this descriptive population study and we consider it more suitable for future studies to pursue.
